# Methods to Enhance Accessibility of Japanese Medical Literature in English Journals

**DOI:** 10.31662/jmaj.2024-0140

**Published:** 2024-10-03

**Authors:** Kosuke Kojo, Bryan J Mathis, Takeshi Yamada, Hiroyuki Nishiyama, Takeshi Machino

**Affiliations:** 1 Tsukuba Clinical Research & Development Organization, University of Tsukuba, Tsukuba, Japan; 2 Department of Urology, Institute of Medicine, University of Tsukuba, Tsukuba, Japan; 3 Clinical Trial Promotion Group, Institute of Medicine, University of Tsukuba, Tsukuba, Japan; 4 Department of Cardiovascular Surgery, Institute of Medicine, University of Tsukuba, Tsukuba, Japan; 5 Department of Gastroenterology, Institute of Medicine, University of Tsukuba, Tsukuba, Japan; 6 Department of Cardiology, Institute of Medicine, University of Tsukuba, Tsukuba, Japan

**Keywords:** Accessibility, AMA Manual of Style, Bibliographic management, Citation guidelines, Cross-lingual citation, Japanese medical literature, Romanization, Vancouver style

## Abstract

To address the challenges of accurately citing Japanese medical literature in English journals, the essential guidelines “Citing Medicine” by the National Library of Medicine were reviewed, focusing on practical adjustments to enhance accessibility. Key proposals include the use of persistent identifiers (Digital Object Identifier, PubMed Identifier, and International Standard Book Number), proper citation of online content, and the inclusion of romanized Japanese article titles. The selection of accessible journal titles and the importance of consistency were also discussed to avoid confusion. Given the significant volume of Japanese medical literature, cross-lingual citation is critical for preventing the isolation of scientific discoveries. These proposals highlight the need for improved citation practices to make Japanese research activities more accessible to the global research community.

## Introduction

The accuracy and completeness of reference lists in academic papers significantly enhance the accessibility of previous research for readers ^(1)^ and demonstrate respect for preceding researchers ^(2)^. Additionally, complete references allow readers to judge for themselves the necessity of consulting the cited works ^(2)^. However, the challenges of accurately citing Japanese medical literature in English journals are rarely discussed. Even native Japanese speakers may struggle to locate original sources, and there are undoubtedly higher barriers for English speakers attempting to access Japanese-only texts.

In this review, we examined the current guidelines and practices for citing Japanese medical literature in English journals. Our lead author authored a paper on the practical use of software for the three-dimensional visualization of CT images available in Japan and reported the findings in the JMA journal ^(3)^. While reviewing technical literature on the use of this software, consideration was given on how to cite Japanese papers in English to enhance accessibility for readers. Drawing from this experience, we highlight key considerations and propose practical methods to improve accessibility in the cross-lingual citing process. These recommendations are particularly relevant to JMA journal readers who are likely to encounter them frequently.

The insights presented here are not novel but are distilled from widely recommended style manuals. By reviewing these guidelines and offering proposals for improvement, we seek to address the existing challenges and enhance the accessibility of Japanese research to the global academic community.

## Style Manuals Referenced

In the medical literature, the *de facto* standard for reference lists is the Vancouver style, which is used by databases such as MEDLINE and PubMed ^(4)^. This style is also endorsed by the International Committee of Medical Journal Editors ^(5)^ and is detailed in “Citing Medicine,” which is published by the National Library of Medicine (NLM) ^(6)^. The AMA Manual of Style, created by the American Medical Association ^(7)^, follows similar principles but includes variations such as italicizing journal names ^(8)^.

The JMA journal, published by the Japan Medical Association, recommends “Citing Medicine” in its instructions for authors ^(9)^, which includes numerous guidelines for citing non-English literature. This review explains practical adjustments based on “Citing Medicine” to improve access to Japanese journal articles, with additional references to the AMA Manual of Style as necessary.

Table 1 presents practical examples of reference styles for citing Japanese literature in English journals, with detailed explanations provided in subsequent sections.

**Table 1.** Example Reference Styles for Citing Japanese Literature in English Journals.

a	Tomoyoshi T. Danshi seishokusen ni kansuru yōgo no rekishiteki hensen―kōgan kara kōgan, soshite seisō e [Etymological confusion in Japanese terms for the testis: past and present]. Hinyokika Kiyo. 1985 Feb;31(2):199-206. Japanese. Cited in: Pubmed; PMID 3893068.
b	Aso Y. Hinyōkikagaku no shōrai [Prospect for urology]. Jpn J Urol. 1989 Jan;80(1)1-2. Japanese. doi: 10.5980/jpnjurol1989.80.1.
c	Kawashima K. Jinsei 100nen jidai, kea o dezainsuru [Designing nursing care in the age of 100 years of life]. J Jpn Acad Gerontol Nurs [Internet]. 2023 Jan [cited 2024 Jun 21];27(2):5-9. Japanese. Available from: https://webview.isho.jp/journal/detail/abs/10.11477/mf.7010200816; https://mol.medicalonline.jp/archive/search?jo=ex3rouka&vo=27&nu=2&st=5; https://mol.medicalonline.jp/en/archive/search?jo=ex3rouka&vo=27&nu=2&st=5
d	Masago T, Morizane S, Hikita K, et al. Sentakuteki dōmyaku soketsu o kokoromita robotto shien jinbubun setsujojutsu no shoki keiken [The initial experience of robot-assisted partial nephrectomy tried selective arterial ischemia]. Jpn J Endourol [Internet]. 2016 Jun [cited 2024 Mar 25];29(1):119-24. Japanese. Available from: https://www.jstage.jst.go.jp/article/jsejje/29/1/29_119/_pdf/; https://mol.medicalonline.jp/archive/search?jo=cs5jjend&vo=29&nu=1&st=119; https://mol.medicalonline.jp/en/archive/search?jo=cs5jjend&vo=29&nu=1&st=119. doi: 10.11302/jsejje.29.119
e	Sonoda T, Kato T. Hinyōkika chiryōgaku [Therapeutic urology] [Internet]. Tokyo (Japan): Igaku Shoin; 1970 Jun [cited 2024 Jun 20]. Japanese. Available from: https://dl.ndl.go.jp/en/pid/12663172. doi: 10.11501/12663172.
f	Ishikawa Y. Shijō genri to Amerika iryō: Nihon no iryō kaikaku no miraikei: Jiyū kyōsō iryō kakusa shakai o ikinuku amerikashiki iryō keiei nyūmon [Introduction to the market mechanism in US medicine]. Tokyo (Japan): Igaku Tsushinsha; 2007 Jul [cited 2024 Jun 20]. Japanese. Available from: https://webview.isho.jp/book/detail/abs/10.32248/9784870583658. ISBN: 978-4-87058-365-8. doi: 10.32248/9784870583658.

## Practical Adjustments to Enhance the Accessibility of Japanese Medical Literature in English Journals

### Indicating persistent identifiers

Since the late 20th century, digital systems have been used to collect information on research activities ^(10)^. Persistent identifiers are essential for accurately and efficiently linking this information ^(11)^. In life sciences and medicine, the PubMed Identifier (PMID), a unique identifier assigned to documents within the PubMed database, has played this role but is gradually being replaced by the Digital Object Identifier (DOI) established in 1997 ^(10)^. DOIs permanently identify digital content, significantly enhancing the utility, visibility, and effect of scholarly works ^(12)^. Adding “https://doi.org/” before the DOI creates a complete Uniform Resource Locator (URL) link ^(13)^. In Japan, the Japan Link Center (JaLC) manages DOI registration, accounting for 3% of the 2.2 billion DOIs registered in 2020 ^(14)^.

According to “Citing Medicine,” PMIDs and DOIs can be included in the note section of most journal article references (Chapter 1, Examples for Notes 70 and 71) ^(6)^. Similarly, the AMA Manual of Style, in its newly established strong recommendations in the 11th edition, states that DOIs should be included whenever possible. Ideally, all content should have a DOI, but, for pre-DOI content, the corresponding PMID should be sought. Table 1a and 1b presents a style example with PMID and DOI, respectively.

### Online content without persistent identifiers

“Citing Medicine” anticipates the existence of online content that lacks persistent identifiers. Such content may be nearly identical to printed journal articles or books. However, “Citing Medicine” emphasizes the importance of not treating these online resources as if they were print materials, as it is crucial to accurately reference the online nature of these resources to maintain proper citation practices. The key is to first gather the necessary details for citing the printed version and then add internet-specific elements (at the beginning of Chapters 22 and 23) ^(6)^. “Citing Medicine” indicates online access by placing “Internet” in square brackets after the title, followed by a period (Chapters 22 and 23, “Type of Medium”) ^(6)^, contrasting with the AMA Manual of Style, which does not specify whether the type of medium is online content or not but requires either a DOI or a URL (DOI preferred) ^(7)^. After location (pagination), start with “Available from:”, followed by the URL. If the URL ends with a slash, add a period; otherwise, no punctuation is required (Chapters 22 and 23, “Availability”) ^(6)^. Multiple URLs can be separated by a space, semicolon, or another space (Chapter 22, Box 67; Chapter 23, Box 59) ^(6)^.

“Citing Medicine” also requires indicating the date of citation after the date of publication because online content is often updated (Chapters 22 and 23, “Date of Citation”) ^(6)^. If available, a DOI may also be included (Chapter 22, Box 69; Chapter 23, Box 61) ^(6)^. This differs from the AMA Manual of Style, which excludes URLs or accessed dates when a DOI is available ^(7)^. Table 1c shows an example with multiple URLs for an article without persistent identifiers. Table 1d provides an example with a DOI and multiple URLs. Table 1e shows the archived content of a book accessible from the National Diet Library’s digital collection, where even older content may have a DOI available, promoting DOI usage ^(15)^.

Additionally, “Citing Medicine” allows the inclusion of an International Standard Book Number (ISBN) at the end of online book references (Chapter 22, Box 71) ^(6)^. ISBNs function as persistent identifiers, uniquely specifying bibliographic records ^(11)^. According to a 2021 report based on a survey of 1,100 biomedical journals and a questionnaire sent to 125 editors or editorial offices, no journals required ISBNs, whereas two-thirds of the respondents considered ISBNs to be important identifiers ^(16)^. ISBNs have been included in Japanese publications since January 1981 ^(17)^. Table 1f presents an example of an online book with both DOI and ISBN.

### Romanized Japanese article titles

**Figure 1.**
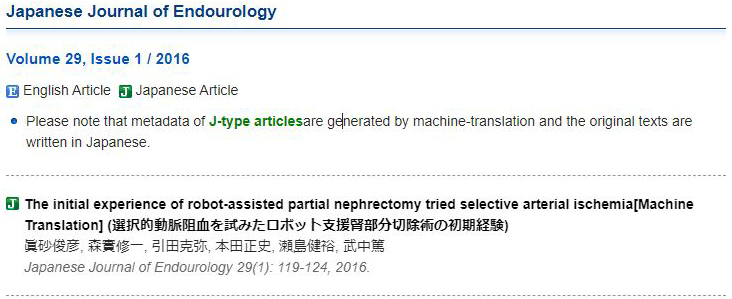
Screenshot of accessing the journal article “Jpn J Endourol. 2016 Jun;29(1):119-24” using the literature database Medical*Online-E (Meteo Inc., Tokyo (Japan)) (retrieved on June 21, 2024): https://mol.medicalonline.jp/en/archive/search?jo=cs5jjend&vo=29&nu=1&st=119. The machine-translated English title, “The initial experience of robot-assisted partial nephrectomy tried selective arterial ischemia [Machine Translation],” is followed by the original Japanese title enclosed in parentheses. This screenshot was cited with the permission of Meteo Inc., in accordance with the requirements of Article 32 of the Japanese Copyright Act.

**Figure 2.**
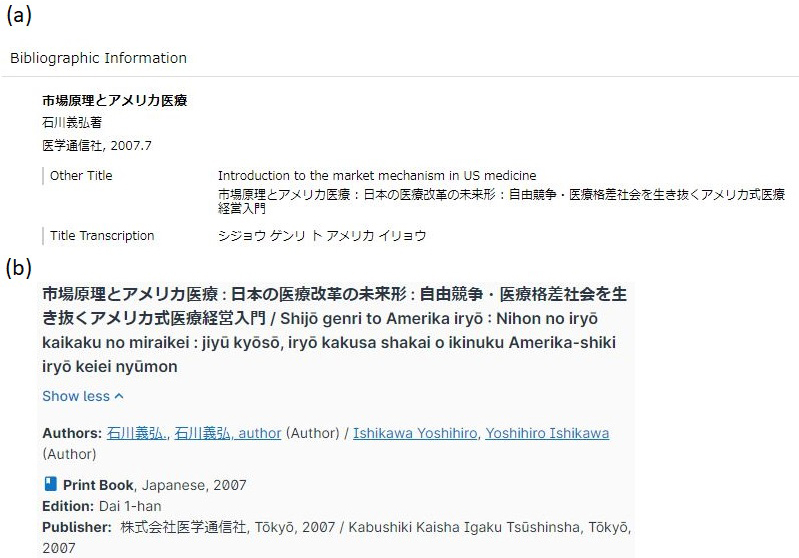
Screenshots of accessing book information with an International Standard Book Number (ISBN) of 9784870583658 using two types of union catalogs. (a) Access result from CiNii (National Institute of Informatics, Tokyo (Japan)) (retrieved on June 21, 2024): https://cir.nii.ac.jp/crid/1130282268695391360?lang=en. The official English title of this book is indicated in the second line of the “Other Title” section. This screenshot was cited with the permission of the National Institute of Informatics in accordance with the requirements of Article 32 of the Japanese Copyright Act. (b) Access result from WorldCat (OCLC Inc. (OH)) (retrieved on June 21, 2024): https://search.worldcat.org/title/180196613. Although accessing this catalog can reveal official English titles for various books, such information is not provided for this particular book. Instead, the romanized title using macrons (ū and ō) “Shijō genri to Amerika iryō: Nihon no iryō kaikaku no miraikei: jiyū kyōsō, iryō kakusa shakai o ikinuku Amerika-shiki iryō keiei nyūmon” is indicated. This screenshot was cited with the permission of the legal department of OCLC, in accordance with the requirements of Article 32 of the Japanese Copyright Act and Section 107 of the United States Copyright Act.

“Citing Medicine” outlines the following specific rules for article titles in languages other than English (Chapter 1, Box 14): Rule 1, translate article titles into English; Rule 2, enclose translated article titles in square brackets; Rule 3, indicate the original language after the location (pagination) with a period; and Rule 4, if possible, place the original or romanized article title before the translation ^(6)^. When no official translation is available, a novel translation must be created. Fortunately, many Japanese medical journals provide official English article titles. However, if such article titles are unavailable, resources like Medical*Online-E (Meteo Inc., Tokyo (Japan)), a Japanese medical literature database, offer machine translations for other journals, which can be helpful (Table 1d and Figure 1). For books, databases like CiNii (National Institute of Informatics, Tokyo (Japan)) or WorldCat (OCLC Inc., Dublin (OH)) may reveal official English article titles (Figure 2).

It is crucial to recognize that the way article titles are presented can significantly affect accessibility and reader comprehension. Rules 2 and 3 are more often omitted than Rule 1 ^(16)^, and Rule 4 (because of official title availability) is seldom followed. However, these rules are also important for indicating non-English content and preventing reader confusion. Unfortunately, many Japanese articles are not found even when searching for their official English article titles in various databases. If such an article is thusly mistaken for an English one, readers may waste time searching; therefore, Rule 3 must be strictly followed, as Rule 2 alone is insufficient. Surprisingly, despite Rule 4 being optional, some guidelines recommend prioritizing the original language title over its translation to clearly indicate that the document is written in a language other than English ^(4)^. Romanized article titles are also beneficial for Japanese speakers who are searching for a Japanese original article title. Table 1 presents examples of romanized Japanese article titles alongside their English translations. It is noteworthy that, as seen in Table 1f, the official English translation of the article title is significantly shorter because of the omission of the Japanese article subtitle, highlighting the importance of including the original language.

One of the challenges in implementing Rule 4 is the romanization of Japanese article titles. Japanese has a long history of multiple, often confusing, romanization systems such as Hepburn and Kunrei, and standardization remains unachieved ^(18)^, which poses a challenge in bibliographic management ^(19)^. “Citing Medicine” considers the American Library Association - Library of Congress Romanization Tables, which are based on the Hepburn system, as reliable authority (Chapter 1, Box 6) ^(6)^. However, these tables use macrons (a type of diacritic, indicated by a horizontal bar written above vowels such as ā, ī, ū, ē, and ō to denote long vowels in Japanese) ^(19), (20)^, which conflict with “Citing Medicine’s” rule to ignore diacritics (Chapter 1, Box 14) ^(6)^. Although macrons are typically not used in the romanization of personal names or well-known place names in Japanese, they are crucial for preserving meaning in general Japanese text. Without macrons, many Japanese words can have altered meanings, leading to potential misunderstandings. This review demonstrates examples with macrons only for article titles, considering technological advancements that make diacritics less problematic. There are various character encoding standards used for electronic communication, among which UTF-8, an international character encoding standard that supports macrons ^(21)^, is widely used across major systems and websites (98.3% as of June 2024) ^(22)^. The AMA Manual of Style and PubMed also accept diacritics, indicating a shift toward their inclusion ^(7)^. Additionally, WorldCat also accepts a romanization system that uses macrons (Figure 2) ^(19)^.

### Accessible journal titles

When citing Japanese journal titles in English papers, two options are available: romanizing the Japanese journal title or using an English (or occasionally Latin) alternative journal title. According to “Citing Medicine,” if the former is selected, the journal title should NOT be abbreviated, whereas, for the latter, specific abbreviation rules apply (Chapter 1, Box 22) ^(6)^. Additionally, “Citing Medicine” allows the inclusion of both romanized and English titles for article titles but does not permit this for journal titles, so one or the other must be selected (Chapter 1, Box 22) ^(6)^.

**Figure 3.**
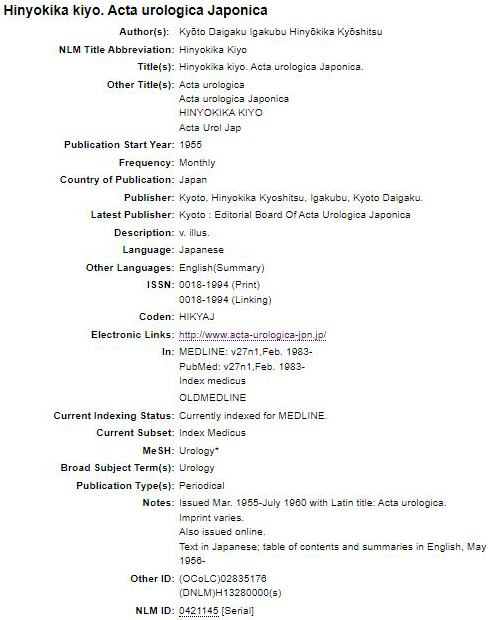
Screenshot of accessing journal information with International Standard Serial Number (ISSN) 0018-1994 using the NLM Catalog (retrieved on June 21, 2024): https://www.ncbi.nlm.nih.gov/nlmcatalog/journals. The primary journal title is indicated as “Hinyokika Kiyo,” the secondary journal title as “Acta Urologica Japonica,” and the abbreviation as “Acta Urol Jap.” This screenshot was cited with the permission of the National Library of Medicine under the requirements of Article 32 of the Japanese Copyright Act and Section 107 of the United States Copyright Act.

For journals indexed in PubMed, the romanized original language journal title is preferred. For example, “Hinyokika kiyo,” which is indexed in PubMed, is the romanized journal title used over its Latin counterpart, “Acta Urologica Japonica,” as it enhances the accessibility of PubMed readers (Table 1a). The NLM Catalog can provide further details, such as the International Standard Serial Number (ISSN) for assigning unique identifiers to periodicals and abbreviations of journal titles, making them easier to reference (Figure 3) (Chapter 1, Box 22) ^(6)^.

**Figure 4.**
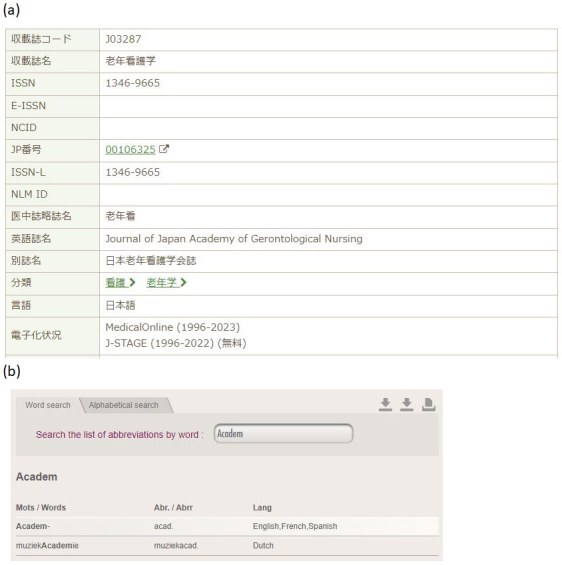
(a) Screenshot of accessing journal information with an International Standard Serial Number (ISSN) of 1346-9665 using the Ichushi Search for Publications function (retrieved on June 21, 2024): https://www.jamas.or.jp/shusaishi/search. The English title “Journal of Japan Academy of Gerontological Nursing” is indicated in the 10th row from the top of the table. This screenshot was cited with the permission of the NPO Japan Medical Abstracts Society in accordance with the requirements of Article 32 of the Japanese Copyright Act. (b) Screenshot of accessing the ISSN International Centre’s List of Title Word Abbreviations (LTWA) (retrieved on June 21, 2024): https://www.issn.org/services/online-services/access-to-the-ltwa/. This shows that the abbreviation for words beginning with “Academ-” is “acad.” This screenshot was cited with the permission of “Le Centre International d´Enregistrement des Publications en Série” in accordance with the requirements of Article 32 of the Japanese Copyright Act and Article L.122-5 of the French Intellectual Property Code.

For journals not indexed in PubMed, the journal title can be freely selected. English (or Latin) journal titles can help non-Japanese readers infer the journal’s subject area because as even Japanese speakers may find it challenging to deduce the original Japanese journal title from its romanized form ^(23)^. Thus, finding English (or Latin) journal titles for nonindexed journals is valuable. Ichushi, a medical literature search service by the Japan Medical Abstracts Society (Tokyo (Japan)), can help identify alternative journal titles (Table 1c and Figure 4a).

“Citing Medicine” also recommends using the ISSN International Centre’s List of Title Word Abbreviations for standard abbreviations (Appendix A) ^(6)^. This resource clarifies abbreviations such as “Journal” to “J,” “Japan” to “Jpn,” “Academ-” to “Acad,” “Gerontolog-” to “Gerontol,” and “Nursing” to “Nurs” (Figure 3b). According to Citing Medicine, abbreviated words should be capitalized, and conjunctions and prepositions should be omitted (Chapter 1, Box 22) ^(6)^. By applying these rules, the journal title “Journal of Japan Academy of Gerontological Nursing” can be abbreviated to “J Jpn Acad Gerontol Nurs” (Table 1c and Figure 4a) (Chapter 1, Box 22) ^(6)^.

Moreover, according to “Citing Medicine,” single-word journal titles should not be abbreviated (Chapter 1, Box 22) ^(6)^. Additionally, if there is a risk of confusion with another journal of the same name, adding the place of publication can clarify this issue (Chapter 1, Box 22) ^(6)^. For example, the journal “Urology,” published by Kagaku Hyoronsha Co., Ltd (ISSN 2435-192X), can be listed as “Urology (Tokyo)” to distinguish it from another journal with the same name published by Elsevier (ISSN 0090-4295). Similarly, the AMA Manual of Style allows for the title to be listed as “*Urology (Tokyo, Japan)*” ^(7)^.

## Discussion

In this review, we have addressed the main challenges and proposed solutions for citing Japanese literature in English journals, following the guidelines of “Citing Medicine.” Specifically, we highlighted the importance of persistent identifiers (DOIs and PMIDs), properly citing online content, including romanized Japanese titles, and selecting accessible journal titles. These strategies enhance the accessibility of Japanese literature and make it more usable for non-Japanese speakers.

Since the Meiji Restoration in the late 19th century, which marked the beginning of Japan’s modernization and westernization, a vast number of Japanese medical journal articles have been published ^(24)^. Considering that Japanese is spoken by approximately 125 million people worldwide and ranks as the eighth most powerful language according to the Power Language Index ^(25)^, which evaluates languages based on geography, economy, business communication, knowledge and media, and diplomacy, it is estimated that the volume of these articles is among the highest per capita in the world. Indeed, the Ichushi Web, a comprehensive bibliographic database of medical literature in Japan, includes 15 million references ^(26)^. Additionally, with 1.23 million members in clinical medicine societies alone, representing one-third of all academic societies in Japan ^(27)^, it is evident that there are a significant number of medical researchers who are native Japanese speakers. Data also show that 40% of researchers whose first language is not English publish their papers in languages other than English ^(28)^. Therefore, Japanese medical researchers often need to cite Japanese journal articles, even when their research findings are published in English. This is especially true for topics deeply rooted in Japanese culture and history, which are frequently recorded only in Japanese ^(29)^.

Cross-lingual citation is crucial for preventing the siloing of scientific discoveries within specific linguistic or cultural groups ^(30)^. Given that English is the *de facto* common language in academia, citations from non-English sources to English are quite common ^(30)^. As the frequency of citing Japanese journal articles in English papers increases, access to such references becomes essential. In the past century, when print media dominated, rare Japanese journals accessible in only a few domestic libraries were often overlooked by international researchers ^(31)^. However, the rise of online journals has dramatically changed this scenario ^(32)^.

By 2021, approximately 30% of Japanese journals offered full-text online links, and this number is steadily growing ^(26)^. Moreover, advances in machine translation have expanded access to Japanese journal articles for non-native speakers ^(33)^. For example, Medical*Online-E offers machine translations of Japanese articles into English, Chinese, and Korean ^(34)^. Additionally, Japanese medical libraries have played a crucial role in supporting evidence-based medicine by providing specialized literature search skills through the Japan Hand Search & Electronic Search Society ^(35)^. Since 1999, the Japan Medical Library Association has encouraged consortium activities to negotiate favorable terms for electronic resources and collaborate with the Japan Pharmaceutical Library Association ^(35)^.

Despite these advances, citing Japanese journal articles in English-language journals remains cumbersome for busy researchers. However, ignoring studies written in languages other than English can introduce bias into meta-analyses ^(36)^. Although reference management software like EndNote (Thomson Reuters, New York (NY)) and Mendeley (Elsevier, Amsterdam (the Netherlands)) is becoming more widespread ^(37)^, and future software updates may ease the burden somewhat, the responsibility for accurate bibliographic information ultimately rests with authors ^(38)^. We hope that this review will help the many diligent researchers in Japan improve their situation.

Publishers also play a critical role in establishing and ensuring accessibility between published articles and the online environment that supports them within the research community ^(39)^. Japanese publishers, in particular, play a significant role in improving accessibility for non-Japanese speakers because they are familiar with unique publishing practices in the Japanese cultural sphere. For example, consider the variations in the common Japanese surname Sato, which can be translated as Sato, Satou, Satoh, or Satoo in romanized form ^(40)^. Romanization serves as a bridge between different languages ^(41)^, but such inconsistencies can create confusion and hinder accurate bibliographic identification. Many Japanese journals now require authors to provide English versions of their names, titles, and abstracts. Given the varying requirements across cultural and academic domains, it may not be practical to radically and strictly unify citation styles ^(42)^. However, as efforts to minimize inconsistencies and confusion in Japanese names and other details demonstrate a commitment to making research accessible to a global audience, such initiatives should require continuous strengthening.

In conclusion, the ongoing efforts of Japanese researchers and publishers to enhance the accessibility of Japanese literature are crucial steps toward contributing to the international academic community. Continued dedication to this cause will ultimately strengthen the effectiveness and integration of Japanese scholarship in the broader academic world.

## Article Information

### Conflicts of Interest

None

### Acknowledgement

The proposals presented are based on a literature review and do not reflect any official opinions of the affiliated institute.

### Author Contributions

KK was primarily responsible for conceptualization, data curation, visualization, and writing the original draft. BM primarily handled writing review and editing. TY, HN, and TM were primarily responsible for supervision. All authors were involved in the interpretation of data for the study, critically revising the study for important intellectual content, final approval of the version to be published and agreement to be accountable for all aspects of the study in ensuring that questions related to the accuracy or integrity of any part of the study are appropriately investigated and resolved. All authors met the ICMJE authorship criteria.

### Approval by Institutional Review Board (IRB)

Not applicable because it does not correspond to “Medical and Health Research Involving Human Subjects.”
